# Competition and equity in NHS specialty training, 2021–2024: longitudinal cohort analysis of protected-characteristic outcomes

**DOI:** 10.1177/01410768261475034

**Published:** 2026-08-12

**Authors:** Dinesh Aggarwal, Meera Roy-Chowdhury, Nicola Xiang, Sharon J Peacock

**Affiliations:** 1 Imperial College London, Faculty of Medicine, London, UK; 2 Wellcome Sanger Institute, Hinxton, Saffron Walden, UK; 3 Jones Lang LaSalle, London, UK; 4 Chelsea and Westminster Hospital, London, UK; 5 Department of Medicine, University of Cambridge, Cambridge, UK; 6 Cambridge University Hospital NHS Foundation Trust, Cambridge, UK

**Keywords:** NHS specialty recruitment, Medical Education, workforce equity, protected characteristics, differential attainment, healthcare workforce, resident doctors

## Abstract

**Objectives:**

To evaluate recruitment equity for NHS specialty training posts between 2021–2024, characterising demographics, quantifying appointment disparities by protected characteristics and assessing temporal trends.

**Design:**

Longitudinal cohort study using national recruitment data via annual Freedom of Information requests.

**Setting:**

National Health Service, UK.

**Participants:**

All medical and surgical specialty training applications to NHS England (2021–2024).

**Main outcome measures:**

Application success by protected characteristics.

**Results:**

Of 214,893 applications, 52,998 (24.7%) were appointed. Annual success rates fell from 12,419/37,971 (32.7%) in 2021 to 13,929/81,189 (17.2%) in 2024. Competitiveness increased but non-UK graduate applications did not reduce successful UK graduate numbers. Gender segregation persisted: surgical specialties had the highest proportion of male applicants, whereas for females this was observed in obstetrics and gynaecology. Female applicants had higher success rates than males (27.5% vs 22.2%; Δ5.3%, 95% CI 4.95–5.70, *p* < 0.001). Pregnant/maternity-leave candidates had lower success (21.3% vs 25.8%, Δ4.5%, 95% CI 3.15–5.82, *p* < 0.001), most markedly in paediatrics (Δ14.8%, 95% CI 8.95–20.58, *p* < 0.001) and surgery (Δ13.7%, 95% CI 7.60–19.82, *p* = 0.008). UK graduates outperformed non-UK graduates (40.7% vs 15.5%; Δ25.2%, 95% CI 24.8–25.6, *p* < 0.001). Adjusted for country of graduation, 15/15 minority ethnic groups had lower odds of success versus White-British, with steepest declines among Black Caribbean and Bangladeshi graduates.

**Conclusions:**

Inequities persist in NHS specialty recruitment. Gender segregation remains and disparities disadvantage pregnant/maternity-leave applicants. Disparities impacting minority ethnic UK graduates have widened. While data constraints preclude adjusting for confounders, these consistent trends require continuous monitoring and targeted interventions to ensure equitable access.

## Introduction

Resident doctors in training comprise approximately 50% of the UK medical workforce, making a fair, effective and unbiased recruitment system for specialty training essential to the future of the NHS.^1^ A medical workforce that reflects patient populations drives better decision-making, reduces bias, fosters innovation and leads to better care.^2,3^ In recent years, increased competition ratios have raised the issue of equity of recruitment and prompted calls from the British Medical Association (BMA) and others for robust, large-scale research to guide targeted interventions which aspire to maintain the diversity of the medical workforce.^4–8^

Securing a national training number is the primary pathway for doctors to complete specialty training and become fully qualified consultants or General Practitioners in the United Kingdom. Applications are made online and mostly processed/organised by a lead NHS England (formally, Health Education England) local office/devolved nation to prevent the need for multiple applications to access posts in different geographical locations. Briefly, the process involves shortlisting of candidates which can include self-assessment or a Multi-Specialty Recruitment Assessment. This is followed with an interview, recommended to be multi-panel and subsequent ranking based on shortlisting and interview scores. If successful, candidates are given an offer of appointment. While designed to be standardised, such staged processes contain points where conscious and unconscious discrimination may occur. Bias against minority ethnic groups during recruitment has been demonstrated in multiple sectors. Meta-analyses of field experiments consistently reveal that job applicants presenting with names associated with ethnic minority groups are less likely to receive a positive response (e.g. call back or job offer) when compared to equally qualified individuals with ethnic majority-sounding names; this has been shown across nations (even if variably) and to be persistent.^9,10^ Within the medical profession, a historical study by Esmail and Everington reported similarly poorer outcomes for matched applicants to UK specialty medicine with Asian names compared with those with English names.^11^ Although current NHS shortlisting protocols have evolved to heavily blind application data, inevitable unblinding occurs during face-to-face or virtual interviews. At this stage, candidates’ ethnicity, gender or maternity status, for example, become apparent and the process becomes vulnerable to biases which can negatively influence panel evaluations.

Between 2019 and 2025, applications for specialty training posts rose from approximately 40,000 to nearly 92,000, pushing the overall competition ratio from 1.9:1 to 7:1.^12^ This bottleneck is exaggerated in certain specialties; the three most competitive specialties included general practice and public health medicine dual training programme (167:1), community sexual and reproductive health (99:1) and cardiothoracic surgery (74:1).^13^ This rise in applications has not been met by a proportionate increase in training posts, leaving many graduates in non-training roles or exiting NHS practice altogether. Moreover, these bottlenecks are likely to disproportionately impact particular demographic groups.

In 2021, our national cross-sectional analysis of 37,971 applications across 58 specialties revealed significant recruitment inequities.^4^ Women secured posts at higher overall rates than men, but we demonstrated that female applications show a lower preference for surgical specialties and radiology. Furthermore, 11 of 15 minority ethnic groups had lower odds of success than White British applicants after adjusting for country of graduation. Finally, although disabled applicants achieved marginally higher success overall, they were unrepresented in over one-third of specialties. This single-year study was, however, limited to three demographic variables in 1 year.^4^

Parallel pressures have exacerbated the difficulties faced by the NHS workforce. Vacancy rates across the entire NHS workforce in England were 6.7%, with greater than 100,000 FTE unfilled roles in March 2025, including nearly 7500 (4.8%) doctor posts.^14^ In 2021, almost 10,000 UK doctors relinquished their licences and up to 44% of senior and 40% of resident doctors report considering leaving, citing pay stagnation, career ‘bottlenecks’, adverse working conditions and training costs.^15^ Furthermore, clinical academic pipelines continue to lose talent at critical transition points, with approximately half of the fully qualified academic doctors not progressing to an academic post in a UK medical school.^16^ Around 65% of the medical school intake is female and approximately 30% are from ethnic minority groups^17,18^; these statistics are not reflected in the post-graduate cohort entering training, a disparity which an equitable recruitment process would aim to address.

Policy frameworks have evolved in response. The NHS Long-Term Workforce Plan and People Plan (2023) committed to doubling medical school places by 2031/2032, increasing GP training posts to 6000 annually, expanding physician and anaesthetic associate workforces and enhancing flexible, less-than-full-time training. Yet, these reforms have not translated into sufficient specialty training posts.^19^ The Public Sector Equality Duty, NHS Workforce Race Equality Standard (WRES) and Disability Equality Standard (WDES) require annual reporting on recruitment, promotion, disciplinary actions and board representation by protected characteristics. Reassuringly, the 2024 WRES reports a rise in minority ethnic senior management representation since 2016, but there remain persistent shortlisting and appointment gaps in a majority of trusts, lower perceptions of fair progression among Black staff (48.8% vs 59.4% White) and ongoing barriers to disability adjustments.^20^ In May 2025, NHS England’s Recruitment Policy Framework further mandated equality impact assessments, transparent selection processes, reasonable adjustments for disability and family circumstances, diverse interview panels and comprehensive data collection on all protected characteristics – these are in line with our recommendations published in 2023.^4,21^

There remains a lack of detailed reporting of disparities in recruitment to specialty training posts in the NHS. To address this gap, we conducted a 4-year, longitudinal analysis of approximately 215,000 applications to medical and surgical specialty training posts in the United Kingdom from 2021 to 2024 by multiple protected characteristics, the largest and most comprehensive study of its kind. Building on previous work, our objectives were to (1) characterise applicant and recruit demographics across an expanded set of protected characteristics; (2) quantify disparities in appointment rates and (3) assess temporal trends to determine whether recent policy interventions have impacted inequities.

## Methods

### Study participants

Data on the gender, pregnancy and maternity leave, ethnicity, disability status, age, sexuality and country of qualification (UK vs non-UK) of applicants to specialty training posts were obtained for each recruitment cycle between 2021 and 2024. To acquire this, the first author submitted annual requests to NHS England under the UK Freedom of Information (FOI) Act 2000, which legally provides public access to data held by national authorities. Demographic details are self-reported, and definitions are provided in Supplemental Methods. NHS England follows the Information Commissioner’s Office code of practice relating to data anonymisation; release of small numbers (<5 in a geographical area/demographic) has therefore been suppressed. Multivariable data were limited due to the risk of identification of participants (Supplemental Methods). Where individual specialities have been grouped, the breakdown of these groups (e.g. surgical) can be found in Supplemental Table 1. All data made available through the FOI request, including specialities where data is incomplete due to low numbers, can be found in Supplemental Data.

Depending on the specialty, length of training can be variable and entry points for candidates can also vary (i.e. some post-graduate resident doctors, previously known as ‘junior doctors’, may enter specialty training in the first year of the respective specialty training programme or may be deemed to have the competencies required to apply at a later stage in the programme); these entry points are denoted by a prefix of ST (speciality training), IMT (internal medicine training) or CT (core training) followed by the year of training at which the specialty training posts is allowing entry.

### Statistical analysis

This national analysis of specialty recruitment examines trends in applicant volume, success rates and disparities across protected characteristics over four consecutive recruitment cycles. All analyses were performed in R (version 4.5.0, R Foundation for Statistical Computing, Vienna, Austria) and Microsoft Excel 2025 (Microsoft Corporation, Redmond, WA, USA). R was chosen because it is an open-source standard in health data science that facilitates fully reproducible code, handles the longitudinal manipulation required for this multi-year dataset and offers packages for advanced regression modelling. Data manipulation and summary tables were created using the tidyverse suite (v2.0.0) and model summaries were tidied with broom (v1.0.10). Figures were generated with ggplot2 (v4.0.0), scales (v1.4.0) and ggeffects (v2.3.1). Descriptive statistics for proportions of successful applications by demographic categories were calculated using the base R prop.test() function, together with Wilson-derived 95% confidence intervals (CIs) via the binom (v1.1-1.1) package. Where 95% CIs do not cross the line of no-effect (i.e. zero in the case of differences in percentages of successful recruitment between categories), the result is statistically significant. When assessing completeness of data to consider the difference in percentages of successful applicants by gender and pregnancy/maternity, data relating to applicants with undisclosed traits are excluded (non-binary is excluded due to the inconsistency in data reporting). Univariable logistic regression models were fitted with glm(outcome ~ predictor, family = binomial, data) to estimate odds ratios (ORs) of success for each covariate. Multivariable models included graduate status (UK vs non-UK qualification) and self-reported ethnic origin (ethnicity model). Gender was included in the multivariable model when considering an interaction between ethnicity and gender. To investigate temporal trends in recruitment outcomes among UK medical graduates, we fitted a logistic regression model with ethnicity and year as predictors, including an interaction term to assess differential changes over time with glm(outcome ~ ethnic origin * year, family = binomial, data). Year was modelled as a continuous linear predictor and comparative models are presented in Supplemental Materials (year as a categorical (factorial) term). Age was modelled as a continuous variable in the primary analysis and evaluated as five-year categories in a separate model as a sensitivity analysis to assess for non-linear relationships. Model selection and comparisons were based on Akaike’s Information Criterion (AIC) and likelihood-ratio tests (ANOVA) and overdispersion and fit were assessed via simulation-based residual diagnostics in DHARMa (v0.4.7). Estimated marginal means and pairwise contrasts on the logit scale were obtained with emmeans (v1.11.2-8) (17). Predicted probabilities across covariate levels were visualised using ggeffects. This study complies with the STROBE reporting guidelines for observational studies in Epidemiology (18).

### Patient and public involvement

Patients or the public were not involved in study design, data collection and analysis, decision to publish or preparation of the manuscript.

## Results

### Cohort overview

There were 214,893 applications across the 2021–2024 recruitment cycles, of which 52,998 (24.7%) were successful. Annual totals rose from 37,971 applications in 2021 (12,419 accepted; 32.7% success) to 81,189 in 2024 (13,929 accepted; 17.2% success) (Supplemental Table 2). These applications spanned 66 specialty posts reclassified into 12 subgroups (Supplemental Table 1); the rising trend in specialty post applications was seen in all specialty groups with a relatively static number of posts (Supplemental Figure 1 and Supplemental Table 3).

### Recruitment by gender

**Figure 1.**
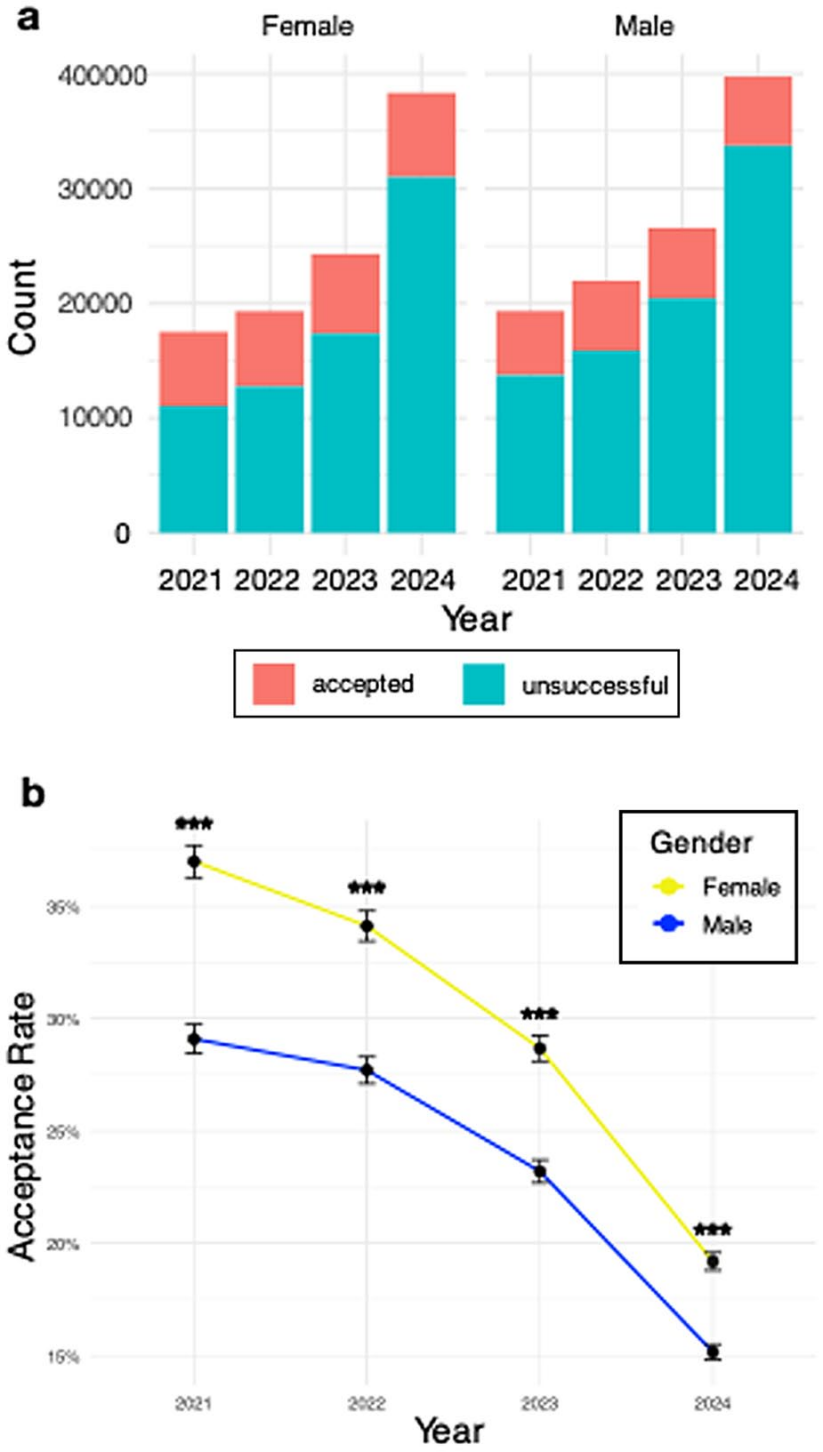
Applications and outcomes for specialty training posts from 2021 to 2024 by gender. (a) Number of successful and unsuccessful applicants to specialty posts by gender from years 2021 to 2024. (b) Acceptance rate of applicants to specialty posts by gender from years 2021 to 2024. Vertical lines represent the 95% confidence intervals around each gender-year acceptance rate, calculated using the Wilson method. *p < 0.05, **p < 0.01, ***p < 0.001 denote statistical significance between female and male acceptance rates within the respective year, calculated via a two-sample test for equality of proportions.

Over the 4 years, 99,518/214,893 (46.3%) of applications were from female candidates and 107,658/214,893 (50.1%) from male candidates. 5552 (2.5%) preferred not to state their gender whilst from 2023, non-binary and ‘other’ categories were introduced. Overall, the difference in percentage of success by females (27,401/99,518, 27.5%) and males (23,907/107,658, 22.2%) was 5.3 percentage points (p.p.) (95% CI = 4.95–5.70 p.p., *p* < 0.001), in favour of females (Figure 1). Female success rates exceeded those of males across all 4 years (*p* < 0.001 for all years) (Figure 1; Supplemental Tables 4–6).

### Recruitment to specialities by gender

**Figure 2.**
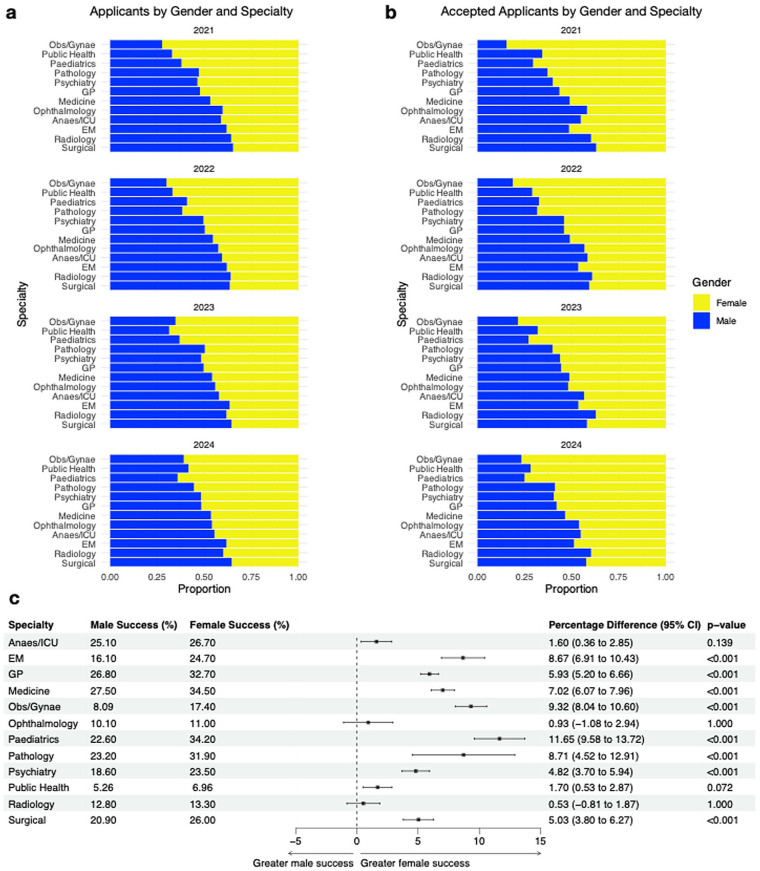
Applications and outcomes for specialty training posts separated into groups of specialties in the 2021–2024 recruitment years by gender. (a) Proportion of applicants to speciality groups by gender by year. (b) Proportion of successful applicants to speciality groups by gender by year. (c) The percentage of successful applicants to specialty posts by gender for 2021–2024, with associated 95% confidence intervals (CIs) derived via the Wald method. *p*-values are adjusted using the Bonferroni method to account for 12 specialty comparisons. Groupings of specialties can be found in Supplemental Table 2.

When examining absolute numbers of applicants and successful applicants by gender to specialty groups, there was an observed segregation by specialty which was consistent across 2021–2024 (Figure 2; Supplemental Table 7 and Supplemental Figure 2). In total, surgical specialities (males, 12,885/19,985, 64.5%) and radiology (males, 6441/10,377, 62.1%) had the highest proportion of male applicants, whereas obstetrics and gynaecology (6579/10,153, 64.8%) and public health (4184/6656, 62.9%) had the highest proportion of female applicants. The gender balance of successful recruits to specialities reflected that of the pool of applicants year on year (Figure 2; Supplemental Figure 2 and Supplemental Data). We examined differences in the proportion of successful applicants to specialty groups; females had a significantly greater percentage of successful candidates compared to males in 10/12 sub-groups but not for Ophthalmology or Radiology (Figure 2).

### Recruitment by pregnancy/maternity status

**Figure 3.**
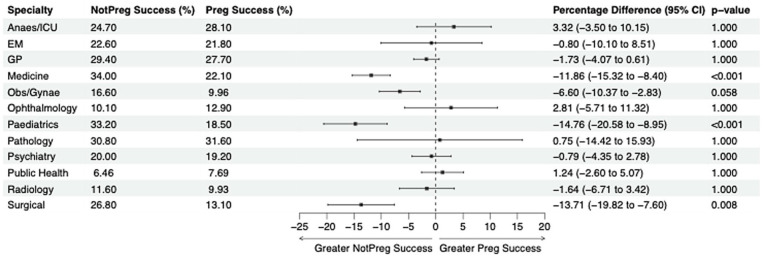
Applications and outcomes for specialty posts by pregnancy and maternity leave status and subspecialty from 2022 to 2024. The percentage of successful applicants to specialty posts by pregnancy and maternity leave status for 2022–2024, with associated 95% confidence intervals (CIs). The 95% CIs for the absolute difference in proportions were calculated using the Wald method. Associated *p*-values have been adjusted for multiple comparisons across the 12 specialty categories using the Bonferroni method. The difference observed in obstetrics and gynaecology (Obs/Gynae), which demonstrated significance prior to correction (unadjusted *p* = 0.005) did not retain statistical significance after adjusting for multiple testing (adjusted *p* = 0.058). Groupings of specialties can be found in Supplemental Table 2.

Pregnancy status is captured by the central application system and remains unknown to the assessment panel. Among applicants with data (2022–2024), the difference in percentage of success by females who were pregnant or on maternity leave (815/3831, 21.3%) and females who were not (19,737/76,619, 25.8%) was 4.5 p.p. (95% CI 3.15–5.82 p.p., *p* < 0.001), in favour of females who were *not* pregnant or on maternity leave. This finding was consistent across all 3 years (Supplemental Figure 3 and Supplemental Table 8). Over the 3-year period, 8/12 specialty groups had lower success rate amongst pregnant females or those on or returning from maternity leave, with 3/12 demonstrating statistically significant differences; paediatrics (14.76 p.p., 95% CI 8.95–20.58, *p* < 0.001), surgery (13.71 p.p., 95% CI 7.60–19.82, *p* = 0.008) and medicine (11.86 p.p., 95% CI = 8.40–15.32, *p* < 0.001) (Figure 3; Supplemental Table 9).

### Recruitment by ethnicity and country of qualification

**Figure 4.**
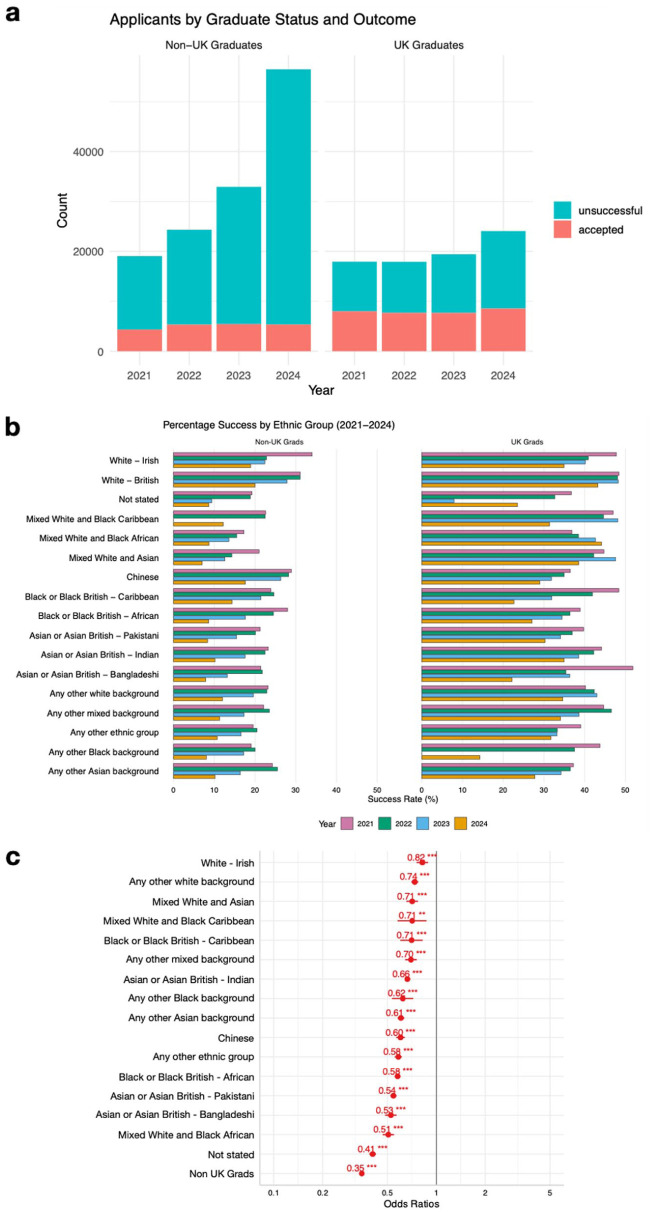
Outcomes from applications to Specialty Posts by Ethnicity and Country of Graduation from 2021 to 2024. (a) Number of successful and unsuccessful applicants to specialty posts by country of qualification from 2021 to 2024 (b) Percentage success to specialty posts by ethnicity and year from 2021 to 2024 (c) Adjusted odds ratios for success by ethnicity compared to White-British where country of qualification (UK vs non-UK) is included as a covariate derived from a multivariable logistic regression model for applicants from 2021 to 2024. Non-medical applicants to public health specialty training have been removed. Odds ratios are presented with 95% confidence intervals. *p < 0.05; **p < 0.01; ***p < 0.001.

UK graduates accounted for 17,939/36,983 (48.5%) of applicants in 2021, falling to 24,082/80,583 (29.9%) in 2024 (Supplemental Table 10). Success rates for UK-qualified applicants declined from 44.5% (7987/17,939) in 2021 to 35.5% (8546/24,082) in 2024, whereas for non-UK graduates fell from 22.8% (4334/19,044) to 9.5% (5345/56,501) (Figure 4; Supplemental Table 10). Overall, the success rate for UK graduates (32,199/79,085, 40.7%) exceeded that of non-UK graduates (20,530/132,610, 15.5%) by 25.2 p.p. (95% CI 24.8–25.6, *p* < 0.001) (Supplemental Table 10).

**Figure 5.**
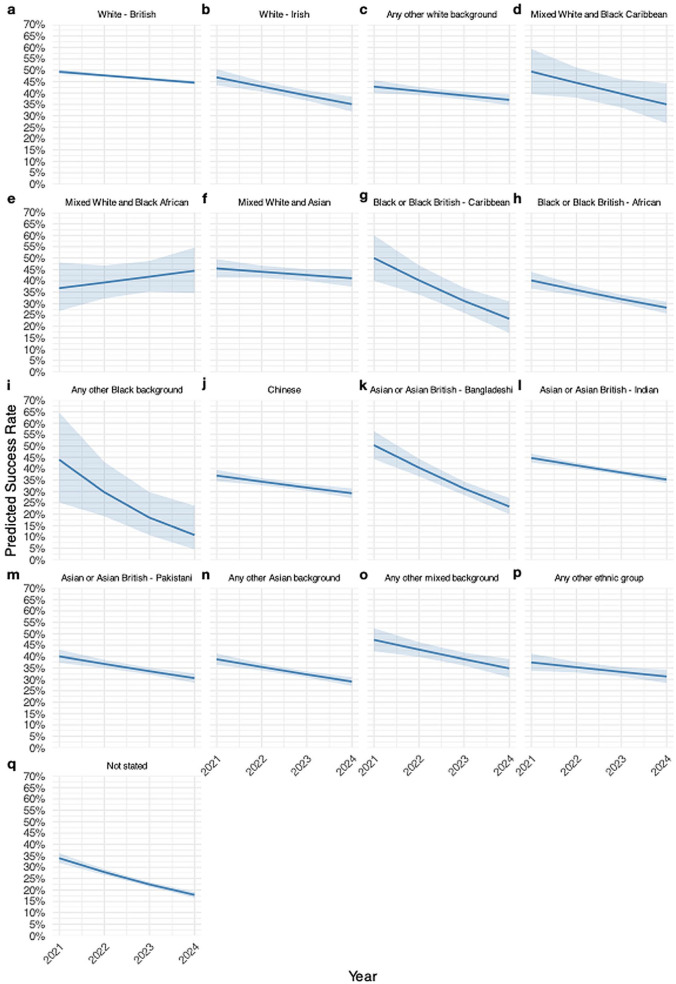
Annual change in predicted success of specialty training success by ethnic origin among UK medical graduates (2021–2024). (a–q) Predicted success rates among UK-graduates from 2021 to 2024, stratified by ethnic origin. Solid lines show model-estimated probabilities from a logistic regression including ethnicity and year interaction. Shaded ribbons represent the corresponding 95% confidence intervals. Each panel represents a separate ethnic group, facilitating comparison of temporal trends.

Logistic regression adjusting for country of graduation found that 15/15 (100%) minority ethnic groups had significantly lower odds of success compared with White-British applicants (Figure 4; Supplemental Table 12 and Supplemental Figures 4–6). Non-UK graduates had an adjusted OR of success of 0.35 (95% CI 0.34–0.36, *p* < 0.001) versus UK graduates. Among UK medical graduates, the odds of a successful specialty application declined each year (OR 0.94/year, 95% CI 0.92–0.96, *p* < 0.001). The decline was greater for 10/15 minority ethnic groups, most notably ‘Any other Black background’ (OR 0.57, 95% CI 0.35–0.90, *p* = 0.02), ‘Asian or Asian British-Bangladeshi’ (OR 0.71, 95% CI 0.63–0.81, *p* < 0.001), Black or Black British-Caribbean (OR 0.72, 95% CI 0.58–0.89, *p* < 0.002) compared with White-British graduates (Figure 5; Supplemental Table 13). For recruitment years 2023 and 2024, multivariable data for ethnicity, gender and country of graduation was available. Using an interaction term between gender and ethnicity and adjusting for graduate status (Ethnicity * Gender + Graduate status), we examined the gender effect variation by ethnicity in a logistic regression model; model assessment with ANOVA showed that there was a non-significant effect (*p* = 0.53).

### Recruitment by disability, age and sexuality

Overall, the difference in percentage of success by those reporting a disability (888/2816, 31.5%) and those who did not (50,393/203,696, 24.7%) was 6.79 p.p. (95% CI 5.05–8.54, *p* < 0.001) in favour of disabled applicants. The lowest percentage of disabled applicants who declared a disability was in 2021 (464/36,418, 1.26%) and the greatest in 2023 (743/50,729, 1.46%). Specialty group data was available for years 2022–2024, during which time Public Health had the highest proportion of self-reported disabled applicants (201/5911, 3.4%) whereas obstetrics and gynaecology had the lowest (76/9023, 0.84%).

For age and sexuality, data were available for years 2022–2024. Applicants ranged from age 23 to 65. Data were complete for 24–53 year olds, and most applicants were between 24 and 40 years old (162,263/176,447, 92.0%) compared to 41–53 year olds (14,184/176,447, 8.0%) (Supplemental Table 14 and Supplemental Figure 8). All older age groups had a statistically significant lower odds ratio for success at application when compared to the 24–29 year group (Supplemental Table 15 and Supplemental Figure 9). There was a consistent decline in success with age (Supplemental Figure 9).

For sexuality, 156,198/176,922 (88.2%) were heterosexual or straight, 2741/176,922 (1.5%) were bisexual, 3240/176,922 (1.8%) were gay or lesbian and 14,743/176,922 (8.3%) were ‘Not Stated or Other’ (Supplemental Table 16). Using ‘Heterosexual or Straight’ as the reference group in a univariable logistic regression model, all other reported categories demonstrated significantly higher odds of success: Bisexual (OR 1.77, 95% CI = 1.63–1.92, *p* < 0.001), Gay or Lesbian (OR 1.65, 95% CI = 1.54–1.78, *p* < 0.001) and Not Stated or Other (OR 1.15, 95% CI = 1.11–1.20, *p* < 0.001) (Supplemental Figure 10 and Supplemental Table 17).

## Discussion

We find rising application numbers with a largely stable intake and therefore an increasing pool of unsuccessful applicants; the number of successful and unsuccessful UK graduate applicants, however, has remained largely static. There is a clear imbalance in applications to specialities by gender, which is consistent across all years. Importantly, for the first time to our knowledge, we show inequities affecting pregnant applicants or applicants on or returning from maternity leave and widening inequities impacting several ethnic minority groups after adjustment for country of qualification. By integrating a broad set of protected characteristics with a longitudinal design, this study provides the first policy-relevant, intersectional analysis of specialty training equity at scale. Our findings will be critical for tracking progress, guiding targeted interventions, informing further research and ensuring that the NHS builds a diverse, resilient medical workforce capable of meeting future healthcare challenges.

Strengths of this study include use of national-level, consecutive-cycle recruitment data and analyses with adjusted models where possible. Comparative analyses of data at sub-specialty levels facilitates targeted interventions. Limitations include reliance on the univariable nature of several descriptive comparisons presented here. This is particularly relevant for age and sexuality comparisons, and the analyses reported should therefore be regarded as hypothesis-generating for those domains. Additionally, missing demographic data such as ‘prefer not to say’ may not be random and instead stem from intentional withholding of information, for example if marginalised applicants anticipate prejudice from disclosure. The absence of candidate-level performance metrics (e.g. portfolio items, shortlisting scores and interview performance) and socio-economic data that could confound observed differences are areas where more research is needed. We have provided an assessment of equitable access by specialty groups, but future work should aim to examine differences at individual specialty level, which is beyond the scope of this work. FOI restrictions prevented adjusting for specialty choice to rule out confounding from differential application rates by demographic groups to highly competitive specialties; the magnitude and persistence of the observed disparities make it unlikely that application skew alone explains our findings. Data on other protected characteristics including religion or belief, marriage or civil partnership and gender reassignment were either not available through an FOI or inconsistently provided and have not been included in this analysis; as a result, we also do not disaggregate data by gender and sex. Furthermore, our findings likely underestimate the true scale of these inequities due to survivor bias, as marginalised groups may self-select out of applying entirely due to prior negative experiences or anticipated systemic barriers. Finally, this work reflects specialty post applications, and we reiterate the need for similar work to be undertaken for allied health professionals and support staff in the NHS.^4^

The total applications to specialty posts increased as a result of increased numbers of medical school places and international applications driven in part by policy changes in skilled worker visas and post-COVID expansion of the Health and Care visa scheme in 2021.^22,23^ Although there remains a large number of UK graduate applicants who are unsuccessful, the decrease in rate of success is largely reflected in non-UK graduates (with a lower odd-ratio for success compared to UK graduates over this study period than our previous report from 2021^4^). The BMA statement that, ‘exponentially increasing competition ratios for speciality training, (are) driven primarily by overseas recruitment’^24^ is not reflected in our data; although *competition ratios* are driven by non-UK graduates, they are not being recruited in increasing numbers to impact the UK graduates. Based, at least in part, on this information, UK graduates were driven to strike action, which highlights the importance of unbiased representation of recruitment data year on year. We do, however, report a steep rise in non-UK graduate applications and the large (but consistent) intake of non-UK graduates in absolute numbers. Historically, this may reflect the equal footing given to international and home medical graduates in the UK specialty selection process – a parity not seen in many other countries. As a result of this rise in overseas applications, the UK parliament has pledged to prioritise UK graduates in future selection processes.^25,26^

The current trend of increasing unsuccessful applications imposes avoidable costs at both system and individual levels. Administratively, the recruitment cycle consumes staff time for eligibility checks, shortlisting, interview panels and subsequent administration. Applicants invest considerable time, fees and travel; repeated rejection represents a high opportunity cost, as these resources could have been directed towards alternative career pathways.^27^ Operational changes that preserve selection validity yet reduce unnecessary workload, such as digital centralised platforms for short-listing and interviewing, automated eligibility screening or validated pre-application gating tests, could materially reduce waste while maintaining fairness.^28–30^

Gender findings require careful interpretation. We show that females are consistently more successful at specialty post applications (*p* < 0.001). However, applications to specialities differ by gender with remarkable consistency over the 4-year study period; this replicates and extends work that we have previously reported and serves to perpetuate gender pay gaps.^4,31^ The overall higher success rate of females is likely to reflect the long-term normalisation process following decades of female under-representation at senior levels.^32^ The stability of gender-specific application and success rates across the study period suggests that observed patterns are not artefactual and underscores the limited progress within our study interval. Surgery and Radiology retain male predominance, whereas paediatrics and obstetrics and gynaecology show female majorities. These patterns are consequential for mentorship, service design, workforce culture and retention, and they justify continued, targeted interventions where gender disparities remain entrenched.^33,34^ Addressing this requires a concerted effort beginning from outreach to schools to mentorship schemes during postgraduate training such as the Women in Surgery Scheme initiated by the Royal College of Surgeons as well as system-level interventions, some of which are discussed below. Furthermore, we have previously provided a framework to help address this important issue.^4^

The finding of lower odds of success for applicants identified as pregnant or on maternity leave is novel and compounds the gender inequity debate. Although additional confounding factors (such as stage of training) require investigation, this effect persisted after stratification by maternal age (above and below age 31, Supplemental Results), which may be considered a confounder, and was present across multiple specialties albeit to differing extents. It is concerning that surgery has both low female attraction and comparatively low success for pregnant applicants. The latter is also observed in female-dominated specialties such as paediatrics and obstetrics and gynaecology (to a lesser extent), likely because the higher number of female applicants makes such statistical trends more apparent. Applicants’ pregnancy or maternity status is captured by the central application system (Oriel) for human resources purposes and is generally not disclosed to shortlisting or interview panels. Therefore, the observed disadvantage is unlikely to represent direct, conscious discrimination by assessors. Instead, these results point towards structural barriers. Rigid portfolio scoring criteria that do not prorate for time out of training, alongside reduced capacity to undertake extracurricular clinical activities or prepare for interviews, inherently disadvantage those on or returning from leave. This reflects the broader, well-documented ‘motherhood penalty’,^31^ compounding the additional personal pressures faced by women in a life-phase where starting a family takes precedence. This suggests a need to adjust selection processes in line with holistic scoring approaches, such as those based on ‘achievement relative to opportunity’.^35,36^ It also highlights the urgency for broader policy reform to address the issue of reduced human capital due to career interruptions which can adversely affect applicants’ scoring in the speciality selection process. Initiatives, such as the National Flexible Working Policies Framework and enhanced leave for pregnancy loss although effective, do not support women *in* work.^37^ It would be prudent to consider investment in, and signposting of, schemes such as on site, out of hours and emergency childcare and better support for paternity care.

We confirm and extend previous work to show ethnic minority applicants have lower success rates than White British applicants after adjusting for country of graduation.^4^ Of particular concern is the pattern of decline in success overall amongst ethnic minority UK graduates when compared to their White British counterpart; this disproportionately affects ethnic minority groups already carrying relative disadvantages (e.g. Bangladeshi and certain Black groups). These findings suggest structural drivers rather than random fluctuation. While NHS shortlisting is blinded to protected characteristics, candidates are unblinded during interviews, introducing a phase where implicit biases may influence panel scoring. Policy measures to address these disparities should target these recruitment vulnerabilities alongside implementation of bias-awareness training for selectors. A potential approach would be to extend blinding into the assessment phase using anonymised situational judgement tests or audio-only interviews; this would require systematic evaluation and feasibility testing. Longer-term capacity-building such as mentoring and targeted preparatory programmes are required to address potential confounders to success such as parental socio-economic status. Transparent and routine publication of disaggregated outcomes by ethnicity and country of qualification is essential to monitor the effect of interventions.

Findings for disability, age and sexual orientation should be interpreted cautiously. Disabled applicants had a higher success rate for specialty posts, but we also observe a markedly lower reporting of disability than the UK working population (23%), which may reflect a hesitancy to declare physical or mental health-related disability. Older applicants had lower unadjusted success rates; however, older age may be confounded by a large representation of non-UK graduates, application bottlenecks and career-breaks, for example. Similarly, observed differences by sexual orientation are likely confounded by cultural and political circumstances resulting in fewer non-UK graduates self-identifying with categorises other than heterosexual. Multivariable modelling that includes country of qualification, specialty and socio-economic indicators is required before substantive conclusions or policy recommendations are drawn with respect to age or sexual orientation.

Implications for national recruitment and policy are fourfold. Firstly, equity protections must be strengthened, including but not limited to routine and transparent equity audits, strengthening measures to anonymise candidates where appropriate, selector training focused on maternity and ethnic bias and targeted support for disproportionately affected groups. Secondly, these data reflect a wider sense of discrimination reported in the NHS workforce; female doctors in surgical specialties report a lack of support and sexism, whereas ethnic minority groups report consistently higher rates of harassment, bullying or abuse from staff at work.^20,34,38^ It is imperative that the NHS fosters a transparent environment where such instances are addressed promptly, as a hostile culture deters top candidates and harms patient care. Thirdly, in order to mitigate for the opportunity cost to unsuccessful candidates, evolving application processes and criteria should be transparent and widely publicised. Finally, the recruitment process should capitalise on the NHS’ digital transformation by implementing automated solutions across centralised digital platforms where feasible, thereby avoiding excessive administrative burden.

In summary, the recruitment system is characterised by rising applicant demand, stable intake of UK graduates and persistent inequities affecting pregnant or maternally absent applicants and several ethnic minority groups. Addressing these issues requires coordinated policy responses spanning capacity planning, process redesign and equity-focused safeguards, accompanied by rigorous transparent evaluation to ensure measurable improvement in fairness and workforce outcomes.

## Supplemental Material

sj-docx-1-jrs-10.1177_01410768261475034 – Supplemental material for Competition and equity in NHS specialty training, 2021–2024: longitudinal cohort analysis of protected-characteristic outcomes Supplemental material, sj-docx-1-jrs-10.1177_01410768261475034 for Competition and equity in NHS specialty training, 2021–2024: longitudinal cohort analysis of protected-characteristic outcomes by Dinesh Aggarwal, Meera Roy-Chowdhury, Nicola Xiang and Sharon J Peacock in Journal of the Royal Society of Medicine

sj-xlsx-1-jrs-10.1177_01410768261475034 – Supplemental material for Competition and equity in NHS specialty training, 2021–2024: longitudinal cohort analysis of protected-characteristic outcomes Supplemental material, sj-xlsx-1-jrs-10.1177_01410768261475034 for Competition and equity in NHS specialty training, 2021–2024: longitudinal cohort analysis of protected-characteristic outcomes by Dinesh Aggarwal, Meera Roy-Chowdhury, Nicola Xiang and Sharon J Peacock in Journal of the Royal Society of Medicine

## Declarations

**Ethical approval:** This study uses data obtained from a Freedom of Information request, that is routinely collected and obtained in line with the Information Commissioner’s Office. Ethical approval is therefore not required. All data relevant to the study are included in the article or uploaded as Supplemental Information and Files.

**Guarantor:** DA.

**Contributorship:** Conceptualisation: DA, MRC; Project administration: DA; Investigation: DA; Data curation: DA; Methodology: DA, MRC; Visualisation: DA; Formal analysis: DA; Writing – original draft preparation: DA; Writing – review and editing: DA, MRC, NX, SJP; Funding acquisition: N/A; Supervision: SJP.

**Provenance:** Not commissioned; peer-reviewed by Julie Morris, Caroline Kamau-Mitchell and Martin McKee.

## References

[1] Department of Health and Social Care. Doctors: question for Department of Health and Social Care. UIN HL15482, tabled on 12 March 2026. See https://questions-statements.parliament.uk/written-questions/detail/2026-03-12/hl15482 (2026).

[2] Gomez LE Bernet P. Diversity improves performance and outcomes. J Natl Med Assoc 2019; 111: 383–392. DOI: 10.1016/j.jnma.2019.01.006. 30765101

[3] Rotenstein LS Reede JY Jena AB. Addressing workforce diversity–a quality-improvement framework. N Engl J Med 2021; 384: 1083–1086. DOI: 10.1056/NEJMp2032224. 33764706

[4] Aggarwal D Roy-Chowdhury M Xiang N Peacock SJ. Applications to medical and surgical specialist training in the UK National Health Service, 2021–2022: a cross-sectional observational study to characterise the diversity of successful applicants. BMJ Open 2023; 13: e069846. DOI: 10.1136/bmjopen-2022-069846. PMC10186087 37076164

[5] Milner A Baker E Jeraj S Butt J. Race-ethnic and gender differences in representation within the English National Health Service: a quantitative analysis. BMJ Open 2020; 10: e034258. DOI: 10.1136/bmjopen-2019-034258. PMC7044822 32060158

[6] Hemmings N Buckingham H Oung C Palmer B. Attracting, supporting and retaining a diverse NHS workforce (2021).

[7] University of Surrey. University of Surrey to lead landmark review into NHS Ethnicity Pay Gap. See https://www.surrey.ac.uk/news/university-surrey-lead-landmark-review-nhs-ethnicity-pay-gap (2025).

[8] British Medical Association. Delivering racial equality in medicine. See https://www.bma.org.uk/ (2022).

[9] Thijssen L van Tubergen F Coenders M Hellpap R Jak S. Discrimination of Black and Muslim minority groups in western societies: evidence from a meta-analysis of field experiments. Int Migr Rev 2022; 56: 843–880. DOI: 10.1177/01979183211045044.

[10] Quillian L Pager D Hexel O Midtboen AH. Meta-analysis of field experiments shows no change in racial discrimination in hiring over time. Proc Natl Acad Sci U S A 2017; 114: 10870–10875. DOI: 10.1073/pnas.1706255114. 28900012 PMC5642692

[11] Esmail A Everington S. Racial discrimination against doctors from ethnic minorities. BMJ 1993; 306: 691–692. DOI: 10.1136/bmj.306.6879.691. 8471921 PMC1677082

[12] Bowie K. Competition ratios: hundreds of doctors compete for some specialty training posts, show ‘scandalous’ NHS data. BMJ 2025; 390: r1981. DOI: 10.1136/bmj.r1981. 40973434

[13] Competition ratios for 2025. See https://medical.hee.nhs.uk/medical-training-recruitment/medical-specialty-training/competition-ratios/2025-competition-ratios (2025).

[14] NHS Digital. NHS Vacancy Statistics, England, April 2015–March 2025, Experimental Statistics. See https://digital.nhs.uk/data-and-information/publications/statistical/nhs-vacancies-survey/april-2015—march-2025-experimental-statistics (2025).

[15] Ferreira T. Escalating competition in NHS: implications for healthcare quality and workforce sustainability. Postgrad Med J 2024; 100: 361–365. DOI: 10.1093/postmj/qgad131. 38204332

[16] Waldock WJ Hughes E Dacre J Sam AH. Is there a sufficient supply of clinical academics for UK medical schools? A retrospective cohort study. BMJ Open 2024; 14: e086211. DOI: 10.1136/bmjopen-2024-086211. PMC11418544 39306352

[17] Ferreira T Collins AM Handscomb A Al-Hashimi D , and the AIMS Collaborative. The role of medical schools in UK students’ career intentions: findings from the AIMS study. BMC Med Educ 2024; 24: 604. DOI: 10.1186/s12909-024-05366-6. PMC11143605 38822263

[18] Brown C Goss C Sam AH. Is the awarding gap at UK medical schools influenced by ethnicity and medical school attended? A retrospective cohort study. BMJ Open 2023; 13: e075945. DOI: 10.1136/bmjopen-2023-075945. PMC10753756 38086586

[19] Specialty-Applications. Specialty training competition ratios. See https://www.specialty-applications.co.uk/competition-ratios/#google_vignette (2025).

[20] NHS England. NHS Workforce Race Equality Standard (WRES): 2024 data analysis report for NHS trusts (2025).

[21] NHS England. Recruitment policy framework. See https://www.england.nhs.uk/publication/recruitment-policy-framework/ (2025).

[22] Khalid S Heather T Palin V Brown OI Drozd M. Navigating UK internal medicine training applications: 10 essential tips. Postgrad Med J 2025; 101: 570–573. DOI: 10.1093/postmj/qgae190. 39724927

[23] GOV-UK. See gov.uk (2021).

[24] Mahase E. Prioritise UK medical graduates for NHS training places, doctors urge. BMJ 2025; 389: r1343 (2025). DOI: 10.1136/bmj.r1343. 40578906

[25] Merritt EC . Competition for specialist training programmes in the NHS: Sector views and the government’s plans for resident doctor training posts. See https://lordslibrary.parliament.uk/competition-for-specialist-training-programmes-in-the-nhs-sector-views-and-the-governments-plans-for-resident-doctor-training-posts/ (2025).

[26] Her Majesty’s Government. The UK’s Points-Based Immigration System (2020).

[27] Best J. The growing bottlenecks in specialty training. BMJ 2023; 382: p1732. DOI: 10.1136/bmj.p1732. 37536730

[28] NHS Employers. Robotic process automation in recruitment. See https://www.nhsemployers.org/case-studies/robotic-process-automation-recruitment (2025).

[29] Patterson F Baron H Carr V Plint S Lane P. Evaluation of three short-listing methodologies for selection into postgraduate training in general practice. Med Educ 2009; 43: 50–57. DOI: 10.1111/j.1365-2923.2008.03238.x. 19140997

[30] Support efficiency for remote multiple mini medical and dental recruitment and selection interviews for NHS nationwide. Eur J Bus Manag Res 2023; 8: 107–113. DOI: 10.24018/ejbmr.2023.8.6.2147.

[31] Jane Dacre D Woodhams C Atkinson C Laliotis I Williams M Blanden J , et al. Mend the gap: The independent review into gender pay gaps in medicine in England. See https://assets.publishing.service.gov.uk/government/uploads/system/uploads/attachment_data/file/944246/Gender_pay_gap_in_medicine_review.pdf (2020).

[32] NHS Digital. Analysis of the representation of women across the hospital and community health services workforce. See https://digital.nhs.uk/data-and-information/find-data-and-publications/supplementary-information/2018-supplementary-information-files/analysis-of-the-representation-of-women-across-the-hospital-and-community-health-services-workforce (2018).

[33] Skinner H Bhatti F. Women in surgery. Bull R Coll Surg Engl 2019; 101: 12–14. DOI: 10.1308/rcsbull.TB2019.12.

[34] Bellini MI Graham Y Hayes C Zakeri R Parks R Papalois V. A woman’s place is in theatre: women’s perceptions and experiences of working in surgery from the Association of Surgeons of Great Britain and Ireland women in surgery working group. BMJ Open 2019; 9: e024349. DOI: 10.1136/bmjopen-2018-024349. PMC6326292 30617103

[35] Lewis M Bhaskaran M Yewers M Love A Latham K Corzo C. Achievement relative to opportunity: beyond academic promotion. See https://sciencegenderequity.org.au/resources/webinar/achievement-relative-to-opportunity-beyond-academic-promotion/ (2023).

[36] National Health | Medical Research Council. NHMRC relative to opportunity policy. See https://www.nhmrc.gov.au/about-us/resources/nhmrc-relative-opportunity-policy (2024).

[37] Department for Business and Trade, Ministry of Housing, Communities and Local Government, Reynolds J and Rayner A. Employment Rights Bill to increase bereavement leave for families who face pregnancy loss. See https://www.gov.uk/government/news/employment-rights-bill-to-increase-bereavement-leave-for-families-who-face-pregnancy-loss (2025).

[38] GMC-UK. Workplace discrimination affects ‘worrying’ number of trainee doctors, GMC survey reveals. See https://www.gmc-uk.org/news/news-archive/nts-2023-workplace-discrimination-affects-worrying-number-of-trainee-doctors-gmc-survey-reveals (2023).

