# Supplementary material for: Competition and equity in NHS specialty training, 2021–2024: longitudinal cohort analysis of protected-characteristic outcomes
Source: J R Soc Med. 2026 Aug 12:01410768261475034. Online ahead of print. doi: 10.1177/01410768261475034 (PMC13473330; doi:10.1177/01410768261475034)
Supplement: Supplementary file 1 [file sj-docx-1-jrs-10.1177_01410768261475034.docx]

| **Speciality** | **Grouping** |
| --- | --- |
| Anaesthetics CT1 | Anaes/ICU |
| Anaesthetics ST4 | Anaes/ICU |
| Intensive Care Medicine ST3 | Anaes/ICU |
| Acute Care Common Stem - Emergency Medicine CT1 ST1 | EM |
| Emergency Medicine ST4 | EM |
| Emergency Medicine ST3 | EM |
| General Practice ST1 | GP |
| Acute Internal Medicine ST4 | Medicine |
| Cardiology ST4 | Medicine |
| Internal Medicine Training CT1 | Medicine |
| Clinical Oncology ST3 | Medicine |
| Combined Infection Training ST3 | Medicine |
| Dermatology ST3 | Medicine |
| Endocrinology and Diabetes Mellitus ST4 | Medicine |
| Gastroenterology ST4 | Medicine |
| Geriatric Medicine ST4 | Medicine |
| Haematology ST3 | Medicine |
| Medical Oncology ST3 | Medicine |
| Neurology ST4 | Medicine |
| Renal Medicine ST4 | Medicine |
| Respiratory Medicine ST4 | Medicine |
| Rheumatology ST4 | Medicine |
| Allergy | Medicine |
| Audio vestibular | Medicine |
| Clinical Genetics | Medicine |
| Clinical Neurophysiology | Medicine |
| Clinical Pharmacology | Medicine |
| Combined infection training ST4 | Medicine |
| General Internal Medicine | Medicine |
| Genitourinary Medicine | Medicine |
| Immunology | Medicine |
| Medical Opthamolgy | Medicine |
| Nuclear Medicine | Medicine |
| Occupational medicine | Medicine |
| Paediatric Cardiology | Medicine |
| Palliative medicine | Medicine |
| Rehab Medicine | Medicine |
| Sports Medicine | Medicine |
| Obstetrics and Gynaecology ST1 | Obs/Gynae |
| Obstetrics and Gynaecology ST3 | Obs/Gynae |
| Community Sexual Health | Obs/Gynae |
| Ophthalmology ST1 | Ophthalmology |
| Ophthalmology St3 | Ophthalmology |
| Paediatrics ST1 | Paediatrics |
| Paediatrics ST3 | Paediatrics |
| Paediatrics ST4 | Paediatrics |
| Histopathology ST1 | Pathology |
| Chemical Pathology | Pathology |
| Diagnostic Neurology | Pathology |
| Paediatric Pathology | Pathology |
| Core Psychiatry Training CT1 | Psychiatry |
| Psychiatry ST4 (all specialties) | Psychiatry |
| Public Health Medicine ST1 | Public Health |
| Clinical Radiology ST1 | Radiology |
| Core Surgical Training CT1 | Surgical |
| General Surgery ST3 | Surgical |
| Otolaryngology ST3 | Surgical |
| Plastic Surgery ST3 | Surgical |
| Trauma and Orthopaedic Surgery ST3 | Surgical |
| Urology ST3 | Surgical |
| Vascular Surgery ST3 | Surgical |
| Cardiothoracic 1 and 3 | Surgical |
| Neurosurgery | Surgical |
| Oral Maxillofacial Surgery 1 and 3 | Surgical |
| Paediatric Surgery | Surgical |
| Thoracic surgery | Surgical |

**Supplementary Table 1: Grouping of individual specialty posts for onward analysis**

Abbreviations: ST, Specialist Trainee (year); CT, Core Trainee (year); ACCS, Acute Care Common Stem.

|  | **2021** | **2022** | **2023** | **2024** | **Total** |
| --- | --- | --- | --- | --- | --- |
| **Total Number of Applications 2024 (all rounds)** | 37971 | 42769 | 52964 | 81189 | 214893 |
| **Total Number of Acceptances 2024 (all rounds)** | 12419 | 13027 | 13623 | 13929 | 52998 |
| **Proportion successful** | 32.7 | 30.5 | 25.7 | 17.2 | 24.7 |

**Supplementary Table 2: Total Number of Applications and Acceptances to Specialty Training Posts between 2021 and 2024 (all rounds).**

| **Specialty** | **Type** | **2021** | **2022** | **2023** | **2024** |
| --- | --- | --- | --- | --- | --- |
| **Anaes/ICU** | **Applicants** | 4564 | 3541 | 4454 | 6862 |
| **Anaes/ICU** | **Accepted** | 1397 | 965 | 1325 | 1325 |
| **EM** | **Applicants** | 1749 | 1922 | 2110 | 2999 |
| **EM** | **Accepted** | 430 | 426 | 407 | 435 |
| **GP** | **Applicants** | 9803 | 11131 | 15171 | 23836 |
| **GP** | **Accepted** | 4484 | 4447 | 4463 | 4466 |
| **Medicine** | **Applicants** | 5554 | 9738 | 9837 | 12202 |
| **Medicine** | **Accepted** | 2093 | 3113 | 3070 | 3207 |
| **Obs/Gynae** | **Applicants** | 1321 | 1507 | 2330 | 4995 |
| **Obs/Gynae** | **Accepted** | 279 | 347 | 392 | 416 |
| **Ophthalmology** | **Applicants** | 673 | 712 | 984 | 1282 |
| **Ophthalmology** | **Accepted** | 98 | 88 | 108 | 89 |
| **Paediatrics** | **Applicants** | 1265 | 1593 | 2217 | 2337 |
| **Paediatrics** | **Accepted** | 468 | 533 | 650 | 561 |
| **Pathology** | **Applicants** | 365 | 329 | 398 | 636 |
| **Pathology** | **Accepted** | 113 | 123 | 123 | 124 |
| **Psychiatry** | **Applicants** | 2693 | 3144 | 4431 | 9825 |
| **Psychiatry** | **Accepted** | 1090 | 1084 | 953 | 1121 |
| **Public Health** | **Applicants** | 959 | 1043 | 1214 | 3440 |
| **Public Health** | **Accepted** | 96 | 93 | 122 | 110 |
| **Radiology** | **Applicants** | 1783 | 2101 | 2926 | 3567 |
| **Radiology** | **Accepted** | 368 | 340 | 342 | 300 |
| **Surgical** | **Applicants** | 4486 | 4532 | 4827 | 6140 |
| **Surgical** | **Accepted** | 1004 | 1116 | 1182 | 1237 |

**Supplementary Table 3: Total Number of Applications and Acceptances to Specialty Training Posts between 2021 and 2024 (all rounds) by subspecialty group.**

| **Year** | **Gender** | **Applicants** | **Accepted Applicants** | **Percentage success** |
| --- | --- | --- | --- | --- |
| **2024** | **Female (inc. trans woman)** | 38354 | 7360 | 19.2 |
|  | **Male (inc. trans man)** | 39767 | 6031 | 15.2 |
|  | **Non-Binary** | 63 | 14 | 22.2 |
|  | **Other** | 57 | 6 | 10.5 |
|  | **Not Stated** | 2948 | 518 | 17.6 |
| **2023** | **Female (inc. trans woman)** | 24313 | 6969 | 28.7 |
|  | **Male (inc. trans man)** | 26586 | 6168 | 23.2 |
|  | **Non-Binary** | 53 | 12 | 22.6 |
|  | **Other** | 20 | <5 | NA |
|  | **Not Stated** | 1992 | 472 | 23.7 |
| **2022** | **Female (inc. trans woman)** | 19328 | 6592 | 34.1 |
|  | **Male (inc. trans man)** | 21965 | 6083 | 27.7 |
|  | **Not Stated** | 1476 | 352 | 23.8 |
| **2021** | **Female (inc. trans woman)** | 17523 | 6480 | 37.0 |
|  | **Male (inc. trans man)** | 19340 | 5625 | 29.1 |
|  | **Not Stated** | 1108 | 314 | 28.3 |

**Supplementary Table 4: Total Number of Applications and Acceptances, and percentage of successful applicants to Specialty Training Posts between 2021 and 2024 (all rounds) by gender.**

| **Gender** | **Year** | **Success rate** | **Standard Error (S.E.)** | **C.I. lower (2.5%)** | **C.I. upper (97.5%)** |
| --- | --- | --- | --- | --- | --- |
| **Female** | 2021 | 0.37 | 0.00365 | 0.363 | 0.377 |
| **Female** | 2022 | 0.341 | 0.00341 | 0.334 | 0.348 |
| **Female** | 2023 | 0.287 | 0.0029 | 0.281 | 0.292 |
| **Female** | 2024 | 0.192 | 0.00201 | 0.188 | 0.196 |
| **Male** | 2021 | 0.291 | 0.00327 | 0.284 | 0.297 |
| **Male** | 2022 | 0.277 | 0.00302 | 0.271 | 0.283 |
| **Male** | 2023 | 0.232 | 0.00259 | 0.227 | 0.237 |
| **Male** | 2024 | 0.152 | 0.0018 | 0.148 | 0.155 |

**Supplementary Table 5: Specialty application success rates by gender and year.** The table shows the calculated success rate, standard error (SE), and the 95% confidence interval (CI) for each group. S.E., standard error; C.I., confidence interval

| **Year** | **Difference in proportions of success (Female vs Male)** | **p value** |
| --- | --- | --- |
| **2024** | 4.02 | <0.0001 |
| **2023** | 5.46 | <0.0001 |
| **2022** | 6.41 | <0.0001 |
| **2021** | 7.9 | <0.0004 |

**Supplementary Table 6: Difference in specialty application success rates by gender and year.** The table shows the calculated difference in success rate between male and female applicants by year, and the associated significance value calculated with a 2-sample test for equality of proportions

|  |  | 2024 | | 2023 | | 2022 | | 2021 | |
| --- | --- | --- | --- | --- | --- | --- | --- | --- | --- |
|  |  | Female | Male | Female | Male | Female | Male | Female | Male |
| Anaes/ICU | All Applicants | 3061 | 3801 | 1878 | 2576 | 1436 | 2105 | 1878 | 2686 |
| Anaes/ICU | All Accepted Applicants | 599 | 726 | 575 | 750 | 401 | 564 | 631 | 766 |
| EM | All Applicants | 1146 | 1853 | 771 | 1339 | 731 | 1191 | 667 | 1082 |
| EM | All Accepted Applicants | 212 | 223 | 189 | 218 | 198 | 228 | 221 | 209 |
| GP | All Applicants | 12299 | 11537 | 7640 | 7531 | 5539 | 5592 | 5128 | 4675 |
| GP | All Accepted Applicants | 2591 | 1875 | 2480 | 1983 | 2405 | 2042 | 2532 | 1952 |
| Medicine | All Applicants | 5668 | 6534 | 4512 | 5325 | 4424 | 5314 | 2601 | 2953 |
| Medicine | All Accepted Applicants | 1716 | 1491 | 1573 | 1497 | 1586 | 1527 | 1068 | 1025 |
| Obs/Gynae | All Applicants | 3044 | 1951 | 1522 | 808 | 1056 | 451 | 957 | 364 |
| Obs/Gynae | All Accepted Applicants | 319 | 97 | 308 | 84 | 282 | 65 | 236 | 43 |
| Ophthalmology | All Applicants | 589 | 693 | 435 | 549 | 303 | 409 | 271 | 402 |
| Ophthalmology | All Accepted Applicants | 41 | 48 | 56 | 52 | 38 | 50 | 41 | 57 |
| Paediatrics | All Applicants | 1500 | 837 | 1400 | 817 | 943 | 650 | 786 | 479 |
| Paediatrics | All Accepted Applicants | 421 | 140 | 474 | 176 | 359 | 174 | 330 | 138 |
| Pathology | All Applicants | 353 | 283 | 198 | 200 | 203 | 126 | 193 | 172 |
| Pathology | All Accepted Applicants | 73 | 51 | 74 | 49 | 84 | 39 | 71 | 42 |
| Psychiatry | All Applicants | 5087 | 4738 | 2289 | 2142 | 1586 | 1558 | 1445 | 1248 |
| Psychiatry | All Accepted Applicants | 667 | 454 | 535 | 418 | 586 | 498 | 654 | 436 |
| Public Health | All Applicants | 2008 | 1432 | 834 | 380 | 698 | 345 | 644 | 315 |
| Public Health | All Accepted Applicants | 79 | 31 | 83 | 39 | 66 | 27 | 63 | 33 |
| Radiology | All Applicants | 1424 | 2143 | 1119 | 1807 | 756 | 1345 | 637 | 1146 |
| Radiology | All Accepted Applicants | 119 | 181 | 127 | 215 | 133 | 207 | 146 | 222 |
| Surgical | All Applicants | 2175 | 3965 | 1715 | 3112 | 1653 | 2879 | 1557 | 2929 |
| Surgical | All Accepted Applicants | 523 | 714 | 495 | 687 | 454 | 662 | 371 | 633 |

**Supplementary Table 7: Applications and successes in sub-specialty group applications by gender and year.**

|  | 2022 | | 2023 | | 2024 | | Total | |
| --- | --- | --- | --- | --- | --- | --- | --- | --- |
| **Pregnancy or Maternity** | **Applicants** | **Accepted** | **Applicants** | **Accepted** | **Applicants** | **Accepted** | **Applicants** | **Accepted** |
| No | 18380 | 6312 | 22527 | 6518 | 35712 | 6907 | 76619 | 19737 |
| Prefer not to say | NA | NA | 609 | 165 | 936 | 204 | 1545 | 369 |
| Yes | 948 | 280 | 1177 | 286 | 1706 | 249 | 3831 | 815 |

**Supplementary Table 8: Total Number of Applications and Acceptances between 2022 and 2024 by Pregnancy/Maternity Leave status.**

| **Applicants** | **Year** | **Specialty** | **Preg/Mat leave** | **Not preg/mat leave** | **Specialty** | **Preg/Mat leave** | **Not preg/mat leave** |
| --- | --- | --- | --- | --- | --- | --- | --- |
| **Total** | 2022 | Anaes/ICU | 38 | 1398 | Paediatrics | 37 | 906 |
| **Accepted** | 2022 | Anaes/ICU | 9 | 399 | Paediatrics | 11 | 348 |
| **Unsuccessful** | 2022 | Anaes/ICU | 29 | 999 | Paediatrics | 26 | 558 |
| **Total** | 2023 | Anaes/ICU | 52 | 1791 | Paediatrics | 77 | 1295 |
| **Accepted** | 2023 | Anaes/ICU | 20 | 544 | Paediatrics | 14 | 452 |
| **Unsuccessful** | 2023 | Anaes/ICU | 32 | 1247 | Paediatrics | 63 | 843 |
| **Total** | 2024 | Anaes/ICU | 81 | 2905 | Paediatrics | 70 | 1403 |
| **Accepted** | 2024 | Anaes/ICU | 19 | 565 | Paediatrics | 9 | 398 |
| **Unsuccessful** | 2024 | Anaes/ICU | 62 | 2340 | Paediatrics | 61 | 1005 |
| **Total** | 2022 | EM | 17 | 714 | Pathology | 11 | 192 |
| **Accepted** | 2022 | EM | 4 | 194 | Pathology | 4 | 80 |
| **Unsuccessful** | 2022 | EM | 13 | 520 | Pathology | 7 | 112 |
| **Total** | 2023 | EM | 31 | 722 | Pathology | 9 | 183 |
| **Accepted** | 2023 | EM | 8 | 176 | Pathology | 4 | 69 |
| **Unsuccessful** | 2023 | EM | 23 | 546 | Pathology | 5 | 114 |
| **Total** | 2024 | EM | 30 | 1087 | Pathology | 18 | 316 |
| **Accepted** | 2024 | EM | 5 | 200 | Pathology | 4 | 64 |
| **Unsuccessful** | 2024 | EM | 25 | 887 | Pathology | 14 | 252 |
| **Total** | 2022 | GP | 366 | 5173 | Psychiatry | 98 | 1488 |
| **Accepted** | 2022 | GP | 155 | 2250 | Psychiatry | 33 | 553 |
| **Unsuccessful** | 2022 | GP | 211 | 2923 | Psychiatry | 65 | 935 |
| **Total** | 2023 | GP | 471 | 6992 | Psychiatry | 130 | 2106 |
| **Accepted** | 2023 | GP | 148 | 2262 | Psychiatry | 24 | 497 |
| **Unsuccessful** | 2023 | GP | 323 | 4730 | Psychiatry | 106 | 1609 |
| **Total** | 2024 | GP | 660 | 11360 | Psychiatry | 271 | 4701 |
| **Accepted** | 2024 | GP | 112 | 2416 | Psychiatry | 39 | 611 |
| **Unsuccessful** | 2024 | GP | 548 | 8944 | Psychiatry | 232 | 4090 |
| **Total** | 2022 | Medicine | 172 | 4252 | Public Health | 53 | 645 |
| **Accepted** | 2022 | Medicine | 45 | 1541 | Public Health | 4 | 62 |
| **Unsuccessful** | 2022 | Medicine | 127 | 2711 | Public Health | 49 | 583 |
| **Total** | 2023 | Medicine | 179 | 4190 | Public Health | 46 | 770 |
| **Accepted** | 2023 | Medicine | 38 | 1503 | Public Health | 7 | 74 |
| **Unsuccessful** | 2023 | Medicine | 141 | 2687 | Public Health | 39 | 696 |
| **Total** | 2024 | Medicine | 232 | 5297 | Public Health | 96 | 1853 |
| **Accepted** | 2024 | Medicine | 46 | 1625 | Public Health | 4 | 75 |
| **Unsuccessful** | 2024 | Medicine | 186 | 3672 | Public Health | 92 | 1778 |
| **Total** | 2022 | Obs/Gynae | 58 | 998 | Radiology | 39 | 717 |
| **Accepted** | 2022 | Obs/Gynae | 8 | 274 | Radiology | 5 | 128 |
| **Unsuccessful** | 2022 | Obs/Gynae | 50 | 724 | Radiology | 34 | 589 |
| **Total** | 2023 | Obs/Gynae | 76 | 1402 | Radiology | 44 | 1037 |
| **Accepted** | 2023 | Obs/Gynae | 13 | 290 | Radiology | 4 | 121 |
| **Unsuccessful** | 2023 | Obs/Gynae | 63 | 1112 | Radiology | 40 | 916 |
| **Total** | 2024 | Obs/Gynae | 127 | 2860 | Radiology | 58 | 1322 |
| **Accepted** | 2024 | Obs/Gynae | 5 | 307 | Radiology | 5 | 107 |
| **Unsuccessful** | 2024 | Obs/Gynae | 122 | 2553 | Radiology | 53 | 1215 |
| **Total** | 2022 | Ophthalmology | 14 | 289 | Surgical | 45 | 1608 |
| **Accepted** | 2022 | Ophthalmology | 4 | 34 | Surgical | 7 | 447 |
| **Unsuccessful** | 2022 | Ophthalmology | 10 | 255 | Surgical | 38 | 1161 |
| **Total** | 2023 | Ophthalmology | 19 | 402 | Surgical | 43 | 1637 |
| **Accepted** | 2023 | Ophthalmology | 4 | 51 | Surgical | 6 | 479 |
| **Unsuccessful** | 2023 | Ophthalmology | 15 | 351 | Surgical | 37 | 1158 |
| **Total** | 2024 | Ophthalmology | 29 | 537 | Surgical | 34 | 2071 |
| **Accepted** | 2024 | Ophthalmology | 0 | 39 | Surgical | 3 | 500 |
| **Unsuccessful** | 2024 | Ophthalmology | 29 | 498 | Surgical | 31 | 1571 |

**Supplementary Table 9: Applications and successes in specialty sub-group applications by maternity/pregnancy status and year.** Where data was missing (<5 and >0), values have been imputed or replaced with ‘4’. Missing values of <5 and >0 only occurred amongst specialty sub-groups in the ‘accepted pregnant/maternity leave applicants’ category and therefore any differences in the favour of the non-pregnant/maternity group are marginally under-estimated.

| **Year** | **Country of Qualification** | **Number of Applicants** | **Successful** | **Percentage (%)** |
| --- | --- | --- | --- | --- |
| **2021** | **UK** | 17939 | 7987 | 44.52 |
|  | **Non UK** | 19044 | 4334 | 22.76 |
| **2022** | **UK** | 17597 | 7619 | 43.30 |
|  | **Non UK** | 24087 | 5313 | 22.06 |
| **2023** | **UK** | 19467 | 8047 | 41.34 |
|  | **Non UK** | 32978 | 5538 | 16.79 |
| **2024** | **UK** | 24082 | 8546 | 35.49 |
|  | **Non UK** | 56501 | 5345 | 9.46 |
| **Total** | **UK** | 79085 | 32199 | 40.71 |
|  | **Non UK** | 132610 | 20530 | 15.48 |

**Supplementary Table 10: Total Number of Applications and Acceptances between 2021 and 2024 by Country of Graduation.**

| **Comparison** | **Percentage difference** | **C.I. lower (2.5%)** | **C.I. upper (97.5%)** | **p-value** |
| --- | --- | --- | --- | --- |
| **Non-UK vs UK graduates (all)** | -24.8 | -24.4 | -25.2 | p<0.001 |
| **Asian or Asian British - Bangladeshi** | -28.7 | -29.9 | -27.4 | p<0.001 |
| **Asian or Asian British - Indian** | -24 | -24.7 | -23.3 | p<0.001 |
| **Asian or Asian British - Pakistani** | -29.5 | -30.2 | -28.8 | p<0.001 |
| **Chinese** | -15.4 | -16.8 | -14.1 | p<0.001 |
| **Any other Asian background** | -24.2 | -25.1 | -23.3 | p<0.001 |
| **Black or Black British - African** | -30.1 | -30.8 | -29.5 | p<0.001 |
| **Black or Black British - Caribbean** | -21.6 | -24.5 | -18.6 | p<0.001 |
| **Any other Black background** | -29.3 | -31.4 | -27.1 | p<0.001 |
| **Mixed White and Asian** | -17.6 | -19.3 | -16 | p<0.001 |
| **Mixed White and Black African** | -32.4 | -33.4 | -31.3 | p<0.001 |
| **Mixed White and Black Caribbean** | -16.5 | -20.8 | -12.2 | p<0.001 |
| **Any other mixed background** | -21.8 | -23.3 | -20.3 | p<0.001 |
| **White - Irish** | -9.03 | -10.9 | -7.12 | p<0.001 |
| **Any other white background** | -21.9 | -22.8 | -21 | p<0.001 |
| **Any other ethnic group** | -28.7 | -29.5 | -27.9 | p<0.001 |
| **Not stated** | -28.5 | -29.3 | -27.7 | p<0.001 |

**Supplementary Table 11: Percentage differences for Non-UK versus UK graduates from 2021 to 2024.** The percentage difference for successful applicants to specialty posts by country of graduation for 2021 to 2024, with associated 95% confidence intervals (CI).

| **Comparison** | **Adjusted Odds Ratios** | **C.I. 2.50%** | **C.I. 97.50%** | **p-value** |
| --- | --- | --- | --- | --- |
| **(Intercept)** | 0.88 | 0.87 | 0.90 |  |
| **Graduate status (Non.UK Grads ~ UK grads)** | 0.35 | 0.34 | 0.36 | p<0.001 |
| **White - Irish** | 0.82 | 0.76 | 0.89 | p<0.001 |
| **Any other white background** | 0.74 | 0.70 | 0.77 | p<0.001 |
| **Mixed White and Black Caribbean** | 0.71 | 0.58 | 0.87 | p<0.001 |
| **Mixed White and Black African** | 0.51 | 0.47 | 0.55 | p<0.001 |
| **Mixed White and Asian** | 0.71 | 0.65 | 0.77 | p<0.001 |
| **Black or Black British - Caribbean** | 0.71 | 0.60 | 0.82 | p<0.001 |
| **Black or Black British - African** | 0.58 | 0.55 | 0.60 | p<0.001 |
| **Any other Black background** | 0.62 | 0.53 | 0.72 | p<0.001 |
| **Chinese** | 0.60 | 0.57 | 0.64 | p<0.001 |
| **Asian or Asian British - Bangladeshi** | 0.53 | 0.48 | 0.57 | p<0.001 |
| **Asian or Asian British - Indian** | 0.66 | 0.64 | 0.69 | p<0.001 |
| **Asian or Asian British - Pakistani** | 0.54 | 0.52 | 0.57 | p<0.001 |
| **Any other Asian background** | 0.61 | 0.58 | 0.64 | p<0.001 |
| **Any other mixed background** | 0.70 | 0.64 | 0.76 | p<0.001 |
| **Any other ethnic group** | 0.58 | 0.56 | 0.61 | p<0.001 |
| **Not stated** | 0.41 | 0.39 | 0.43 | p<0.001 |

**Supplementary Table 12: The effect of ethnicity on success at applications to specialty posts amongst UK graduates relative to White British applicants after adjusting for country of graduation from 2021 to 2024. C.I., confidence interval.** Multivariable logistic regression model comparing success of application to Specialty Training Posts by minority ethnic groups to White British applicants where Country of Qualification (UK versus Non-UK) is included as a covariate. Non-Medical applicants to Public Health Specialty Training have been removed. Odds ratios are presented with 95% Confidence Intervals.

| **Term** | **estimate** | **S.E** | **statistic** | **p.value** | **C.I 2.5%** | **C.I 97.5%** |
| --- | --- | --- | --- | --- | --- | --- |
| **Year** | 0.938*** | 0.00929 | -6.86 | <0.001 | 0.921 | 0.955 |
| **White - Irish** | 0.905* | 0.0402 | -2.49 | 0.013 | 0.836 | 0.979 |
| **Any other white background** | 0.983 | 0.0309 | -0.542 | 0.59 | 0.925 | 1.04 |
| **Mixed White and Black Caribbean** | 0.874* | 0.107 | -1.26 | 0.02 | 0.708 | 1.08 |
| **Mixed White and Black African** | 1.19 | 0.118 | 1.44 | 0.15 | 0.942 | 1.5 |
| **Mixed White and Asian** | 1 | 0.0444 | 0.112 | 0.91 | 0.921 | 1.1 |
| **Black or Black British - Caribbean** | 0.717** | 0.109 | -3.06 | 0.002 | 0.578 | 0.885 |
| **Black or Black British - African** | 0.891** | 0.0393 | -2.94 | 0.003 | 0.825 | 0.962 |
| **Any other Black background** | 0.574* | 0.238 | -2.33 | 0.02 | 0.35 | 0.901 |
| **Chinese** | 0.948 | 0.0305 | -1.75 | 0.08 | 0.893 | 1.01 |
| **Asian or Asian British - Bangladeshi** | 0.714*** | 0.0613 | -5.49 | <0.001 | 0.633 | 0.805 |
| **Asian or Asian British - Indian** | 0.934** | 0.0221 | -3.08 | 0.002 | 0.894 | 0.975 |
| **Asian or Asian British - Pakistani** | 0.926* | 0.0311 | -2.47 | 0.014 | 0.871 | 0.984 |
| **Any other Asian background** | 0.92** | 0.0283 | -2.94 | 0.003 | 0.87 | 0.973 |
| **Any other mixed background** | 0.896* | 0.0528 | -2.09 | 0.037 | 0.807 | 0.993 |
| **Any other ethnic group** | 0.972 | 0.0413 | -0.684 | 0.49 | 0.896 | 1.05 |
| **Not stated** | 0.799*** | 0.0268 | -8.36 | <0.001 | 0.758 | 0.842 |

**Supplementary Table 13: The effect of ethnicity on success at applications to specialty posts amongst UK graduates relative to White British applicants year on year from 2021 to 2024. S.E., standard error; C.I., confidence interval.** A logistic regression model is fitted with variation of success at application between ethnic groups by year in a sub-group population of UK graduates only. White-British are used as the reference group. This is measured by including an interaction term between the ethnicity and year, modelled as a continuous trait. Model estimates represent the annual change in predicted probability of success for UK graduates, stratified by ethnic group. Probabilities are presented with 95% (2.5% - 97.5%). ** p<0.05   ** p<0.01   *** p<0.001.*

| **Age group** | **Total applicants** | **Total successful applicants** | **Percentage success (%)** |
| --- | --- | --- | --- |
| **24–29** | 72747 | 19808 | 27.2 |
| **30–34** | 63928 | 14147 | 22.1 |
| **35–39** | 25588 | 4523 | 17.7 |
| **40–44** | 9592 | 1453 | 15.1 |
| **45–49** | 3645 | 482 | 13.2 |
| **50–53** | 947 | 119 | 12.6 |

**Supplementary Table 14: Age group counts and success from 2022-2024**

| **Age Group** | **Odds Ratio** | **C.I. 2.5%** | **C.I. 97.5%** | **P-value** |
| --- | --- | --- | --- | --- |
| Intercept | 0.943 | 0.941 | 0.946 | p<0.001 |
| 30–34 | 0.751 | 0.732 | 0.77 | p<0.001 |
| 35–39 | 0.552 | 0.533 | 0.573 | p<0.001 |
| 40–44 | 0.458 | 0.432 | 0.485 | p<0.001 |
| 45–49 | 0.395 | 0.358 | 0.435 | p<0.001 |
| 50–53 | 0.361 | 0.296 | 0.437 | p<0.001 |

**Supplementary Table 15: Effect of age on success at specialty post applications from 2022 to 2024.** Univariable logistic regression model fit to data with age modelled as continuous (top row) and in groups. For age group analysis, 24-29 year old age group is used as the reference point. C.I., confidence intervals are presented with 95% (2.5% - 97.5%). ** p<0.05   ** p<0.01   *** p<0.001.*

| **Sexuality** | **Year** | **Applicants** | **Accepted** | **Unsuccessful** | **Percentage successful (%)** |
| --- | --- | --- | --- | --- | --- |
| **Bisexual** | 2024 | 1276 | 374 | 902 | 29.3 |
| **Bisexual** | 2023 | 812 | 307 | 505 | 37.8 |
| **Bisexual** | 2022 | 653 | 245 | 408 | 37.5 |
| **Gay or Lesbian** | 2024 | 1358 | 371 | 987 | 27.3 |
| **Gay or Lesbian** | 2023 | 1038 | 348 | 690 | 33.5 |
| **Gay or Lesbian** | 2022 | 844 | 327 | 517 | 38.7 |
| **Heterosexual or Straight** | 2024 | 72370 | 11912 | 60458 | 16.5 |
| **Heterosexual or Straight** | 2023 | 46834 | 11824 | 35010 | 25.2 |
| **Heterosexual or Straight** | 2022 | 36994 | 11200 | 25794 | 30.3 |
| **Not Stated or Other** | 2024 | 6185 | 1272 | 4913 | 20.6 |
| **Not Stated or Other** | 2023 | 4280 | 1144 | 3136 | 26.7 |
| **Not Stated or Other** | 2022 | 4278 | 1255 | 3023 | 29.3 |

**Supplementary Table 16: Applicants numbers and success by sexuality from 2022-2024**

| **Sexuality** | **Odds Ratio** | **Confidence Interval 2.5%** | **Confidence Interval 97.5%** | **P-value** |
| --- | --- | --- | --- | --- |
| **Bisexual** | 1.77 | 1.63 | 1.92 | p<0.001 |
| **Gay or Lesbian** | 1.65 | 1.54 | 1.78 | p<0.001 |
| **Not Stated or Other** | 1.15 | 1.11 | 1.20 | p<0.001 |

**Supplementary Table 15: Odds ratio of success by sexuality compared to “Heterosexual or Straight” for all applicants between 2022 and 2024.** Univariable logistic regression model fit to data with sexuality modelled with Heterosexual category as the reference group. C.I., confidence intervals are presented with 95% (2.5% - 97.5%).

**Supplementary Figures**

**Supplementary Figure 1: Trends in application numbers and acceptances to specialty posts by specialty groups from 2021 to 2024**

**Supplementary Figure 2: Applicant and Success counts by gender and specialty to specialty posts from years 2021-2024.**

**Supplementary Figure 3: Proportion of successful and unsuccessful candidates for specialty posts by pregnancy and maternity leave status amongst females from 2022 to 2024.** Candidates self reported if they were pregnant, on maternity leave, or within six months of pregnancy and had returned from maternity leave.

**Supplementary Figure 4: Success rate by ethnicity and country of graduation to specialty posts by year, from 2021 to 2024.** Success rate represents percentage of successful candidates in each sub-category.

**Supplementary Figure 5: Counts of successful and unsuccessful candidates by ethnicity and country of graduation to specialty posts by year, from 2021 to 2024.**

**Supplementary Figure 6: Number of successful candidates by ethnicity and country of graduation to specialty posts by year, from 2021 to 2024.**

**Supplementary Figure 7: Annual change in predicted success of Specialty Training success by ethnic origin among UK medical graduates (2021–2024).** Predicted success rates among UK‐graduates from 2021 to 2024, stratified by ethnic origin. Solid lines show model‐estimated probabilities from a logistic regression including ethnicity and year interaction, where year is treated as a factorial term. Results are consistent with modelling year as a linear term. Shaded ribbons represent the corresponding 95% Confidence Intervals. Each panel represents a separate ethnic group, facilitating comparison of temporal trends.

**Supplementary Figure 8: Number of successful and unsuccessful candidates by age to specialty posts from 2022 to 2024.** (a) stacked absolute counts of successful and unsuccessful applicants by age (b) proportion of successful and unsuccessful applicants by age

**Supplementary Figure 9: Predicted success to specialty posts by age for years 2022 to 2024.** (a) Age modelled in a logistic regression model as a continuous and an interaction with year. (b) Age modelled in a logistic regression model as a categorical variable with age binned in groups, and an interaction with year.

**Supplementary Figure 10: Odds ratio for success by sexuality compared to “Heterosexual or Straight” category for applicants between 2022-2024.** Unadjusted Odds ratios for success by sexuality when compared to “Heterosexual or Straight” applicants derived from a logistic regression model for applicants from 2022 to 2024

**Supplementary Methods**

Options for candidates to self-report included:

- Gender: ‘Male’, ‘Female’, and ‘Not Stated’. Non-Binary and Other options were included from 2023 onwards – in line with our previous recommendations.
- Ethnicity: ‘White - British ‘, ‘Asian or Asian British - Bangladeshi’, ‘Asian or Asian British - Indian’, ‘Asian or Asian British - Pakistani’, ‘Chinese’, ‘Any other Asian background’, ‘Black or Black British - African’, ‘Black or Black British - Caribbean’, ‘Any other Black background’, ‘Mixed White and Asian’, ‘Mixed White and Black African’, ‘Mixed White and Black Caribbean’, ‘Any other Mixed background’, ‘White - Irish’, ‘Any other White background’, ‘Any other ethnic background’, and ‘Not stated’.
- Disability: ‘Yes’ (i.e. applicant has a disability), ‘No’, and ‘Not stated’. Data pertaining to the category or type of disability was not available for analysis.
- Pregnant, on maternity leave, or within six months of pregnancy: “Yes”, “No”, “Not Stated”
- Sexuality: “Bisexual”, “Gay or Lesbian”, “Heterosexual or Straight”, “Not Stated”. “Other sexual orientation not listed” was included from 2023 onwards.
- Further details on the application process for individual specialties in the UK can be found here, https://medical.hee.nhs.uk/medical-training-recruitment/ (16).

Access to data:

Demographic details are self-reported, and definitions are provided in Supplementary Methods. NHS England follows the Information Commissioner's Office code of practice relating to data anonymisation; release of small numbers (<5 in a geographical area/demographic) has therefore been suppressed. Multivariable data were limited due to the risk of identification of participants.

Following an initial analysis of 2021-2022 data (Aggarwal et al.)^1^, we progressively broadened our Freedom of Information (FOI) requests. Because FOI teams can refuse overly burdensome queries, and to minimise data suppression, our requests had to be precise. As our understanding of the underlying database evolved, we optimised our approach to FOI requests to maximise data yield. Consequently, more linked variables became available in recent years to test targeted hypotheses (e.g. when considering an interaction between gender and ethnicity). The following linked data were successfully obtained:

- Total number of applicants and acceptances aggregated across all specialities for 2021 to 2024 recruitment years (unlinked).
- Total number of applicants and acceptances aggregated across all specialities (unlinked) by the following characteristics for:
  - Gender (years 2021 to 2024)
  - Pregnancy and maternity leave (years 2022 to 2024)
  - Ethnic origin (years 2021 to 2024)
  - Disability (years 2021 to 2024)
  - Age by year (years 2022 to 2024)
  - Sexuality (years 2022 to 2024)
  - Country of Graduation (UK vs Non-UK) (years 2021 to 2024)
- The total number of applicants and acceptances aggregated across all specialities by Ethnicity linked to Country of Graduation (UK vs Non-UK) for 2021 to 2024 recruitment years.
- The total number of applicants and acceptances aggregated across all specialities by Ethnicity linked to Country of Graduation (UK vs Non-UK) and Gender for 2023 and 2024 recruitment years.
- Data on Pregnancy and maternity leave, for females only, split by those above the age of 31 and those below the age of 31 for the 2024 recruitment year.
- Specialty grouping data (Supplementary Table 1) for applications and acceptances by:
  - Gender (years 2021 to 2024)
  - Pregnancy and maternity leave (years 2022 to 2024)
  - Disability (years 2021 to 2024)

**Supplementary Results**

Age stratified pregnancy and maternity status related data was available for the 2024 recruitment year. This was requested to examine whether age was a likely confounder in the differences in success by pregnancy and maternity status observed. The difference in percentage of success by females who were pregnant or on maternity leave under 31 (71/616, 11.5%) and females who were pregnant or on maternity leave over 31 (178/1,090, 16.3%) was 4.8 p.p. (95% CI= 1.33-8.27 p.p., p=0.009), in favour of females who were over 31. Both figures are below the percentage success of non-pregnant females (6,907/35,712, 19.3%).

1	Aggarwal, D., Roy-Chowdhury, M., Xiang, N. & Peacock, S. J. Applications to medical and surgical specialist training in the UK National Health Service, 2021-2022: a cross-sectional observational study to characterise the diversity of successful applicants. *BMJ Open* **13**, e069846 (2023). <https://doi.org/10.1136/bmjopen-2022-069846>
